# Fc**γ** receptor–mediated cross-linking codefines the immunostimulatory activity of anti-human CD96 antibodies

**DOI:** 10.1172/jci.insight.158444

**Published:** 2022-10-10

**Authors:** Anne Rogel, Fathima M. Ibrahim, Stephen M. Thirdborough, Florence Renart-Depontieu, Charles N. Birts, Sarah L. Buchan, Xavier Preville, Emma V. King, Aymen Al-Shamkhani

**Affiliations:** 1 Centre for Cancer Immunology, School of Cancer Sciences, Faculty of Medicine, University of Southampton, Southampton, United Kingdom.; 2 Blink Biomedical, Lyon, France.; 3 Talix Therapeutics, Leuven, Belgium.

**Keywords:** Immunology, Cancer immunotherapy, Costimulation, T cells

## Abstract

New strategies that augment T cell responses are required to broaden the therapeutic arsenal against cancer. CD96, TIGIT, and CD226 are receptors that bind to a communal ligand, CD155, and transduce either inhibitory or activating signals. The function of TIGIT and CD226 is established, whereas the role of CD96 remains ambiguous. Using a panel of engineered antibodies, we discovered that the T cell stimulatory activity of anti-CD96 antibodies requires antibody cross-linking and is potentiated by Fcγ receptors. Thus, soluble “Fc silent” anti-CD96 antibodies failed to stimulate human T cells, whereas the same antibodies were stimulatory after coating onto plastic surfaces. Remarkably, the activity of soluble anti-CD96 antibodies was reinstated by engineering the Fc domain to a human IgG1 isotype, and it was dependent on antibody *trans*-cross-linking by FcγRI. In contrast, neither human IgG2 nor variants with increased Fcγ receptor IIB binding possessed stimulatory activity. Anti-CD96 antibodies acted directly on T cells and augmented gene expression networks associated with T cell activation, leading to proliferation, cytokine secretion, and resistance to Treg suppression. Furthermore, CD96 expression correlated with survival in HPV^+^ head and neck squamous cell carcinoma, and its cross-linking activated tumor-infiltrating T cells, thus highlighting the potential of anti-CD96 antibodies in cancer immunotherapy.

## Introduction

The clinical success of agents targeting immune checkpoint receptors, such as CTLA-4 and PD-1, has demonstrated that the immune system is a bona fide and key therapeutic target for the treatment of cancer. Despite the unprecedented durable antitumor responses seen in a subset of patients, the majority of patients fail to respond to these treatments or develop resistance after the initial response (1). This has galvanized the search of additional immune checkpoint receptors that could be targeted to extend the benefit of immunotherapy to the wider population (2). One such receptor that has recently received attention is CD96, also known as T cell–activated late expression (TACTILE). CD96 is a type I transmembrane protein comprising an extracellular region that consists of 3 immunoglobulin superfamily (IgSF) domains followed by an O-glycosylated stalk region (3, 4). The cytoplasmic domain of CD96 contains a conserved short basic/proline-rich motif, which typically associates with SH3-domain-containing proteins, followed by a single immunoreceptor tyrosine-based inhibitory motif (ITIM). In addition, a YXXM motif similar to that found in CD28 and ICOS is present in human but not mouse CD96. Expression of CD96 is limited to immune cells, primarily T cells, NK cells, and NKT cells, and is upregulated following T cell activation (3, 5). Two isoforms of CD96 that differ in the sequence of the second IgSF domain exist as a result of alternative splicing, with the shorter isoform (CD96v2) being the predominant form expressed in human primary cells (6).

CD96 shares an ability to bind proteins from the nectin and nectin-like family with 2 other IgSF receptors, namely T cell immunoreceptor with Ig and ITIM domains (TIGIT) and CD226 (also known as DNAX accessory molecule 1 [DNAM-1]). While TIGIT and CD226 bind to CD155 (necl-5) and CD112 (nectin-2), CD155 is the only known ligand for CD96 in humans (7). CD155 is weakly expressed on a variety of cells, including immune, epithelial, and endothelial cells, and is upregulated on cancer cells (8, 9). TIGIT and CD226 function as inhibitory and activating receptors, respectively, while both inhibitory and stimulatory functions have been ascribed to CD96. Initial studies demonstrated that engagement of CD96 stimulates human NK cell–mediated lysis of P815 cells in redirected killing assays, albeit less efficiently than CD226 (10, 11). Furthermore, unlike CD226, CD96 was dispensable for killing of CD155-expressing tumor cells, suggesting that the stimulatory effect of CD226 is dominant (12, 13). In contrast, studies in mice showed that CD96 deficiency results in an exaggerated NK cell–mediated IFN-γ production and resistance to carcinogenesis and experimental lung metastases (14), indicating that CD96 functions as an inhibitory receptor in murine NK cells. Additional studies employing anti-CD96 antibodies provided further support for targeting this pathway as a strategy to treat cancer (14, 15); however, the findings were confounded by the observation that anti-CD96 antibodies need not block the CD155-CD96 interaction to exert their antimetastatic effect (16). More recently, Chiang et al. (17) showed that genetic ablation or antibody blockade of CD96 rendered murine CD8^+^ T cells less responsive and, conversely, that anti-CD96 antibody presented on microbeads promoted T cell proliferation. Antibodies have the capacity to induce receptor clustering dependent on coengagement of Fcγ receptors (FcγRs), and this property has been exploited for the development of agonistic immunostimulatory antibodies that target costimulatory TNF receptor superfamily members (18–20).

Here, we have addressed whether FcγR cross-linking potentiates the activity of anti-human CD96 antibodies. Through Fc domain engineering, we have identified the human IgG1 isotype as a key determinant that codefines the activity of anti-CD96 antibodies. We show that anti-CD96 antibodies costimulate the proliferation of human peripheral CD4^+^ and CD8^+^ T cells and enhance cytokine production in an isotype- and FcγRI-dependent manner. Costimulation by anti-CD96 antibodies was effective in countering suppression by Tregs and in inducing the proliferation of tumor-infiltrating T cells (TILs). RNA-Seq analysis following CD96 costimulation revealed upregulation of multiple gene networks associated with T cell proliferation and effector function. These results inform the design of immunostimulatory anti-CD96 antibodies for the reinvigoration of anticancer T cells.

## Results

### Immobilized and FcγR cross-linked anti-CD96 antibodies promote human T cell proliferation.

**Figure 1:**
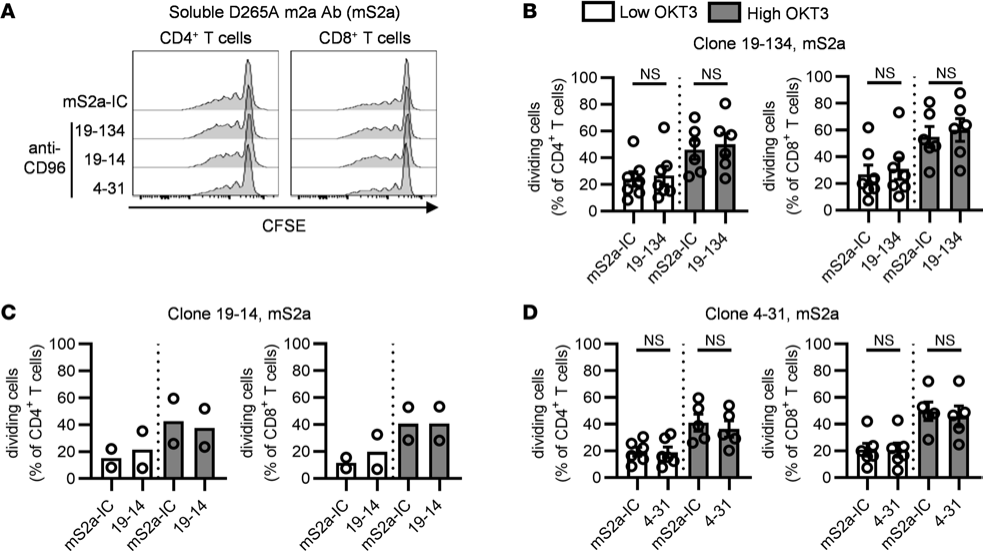
The CD155-CD96 interaction is not critical for T cell proliferation in human PBMCs. CFSE-labeled PBMCs from healthy donors (HDs) were stimulated with 2 concentrations of OKT3 for 4 days in the presence of soluble D265A m2a (mS2a) anti-CD96 mAbs (clones 19-134, 19-14, and 4-31) or a matching isotype control (mS2a-IC). The proportion of dividing cells among CD4^+^ and CD8^+^ T cells was analyzed by flow cytometry. (**A**) Representative examples of CFSE dilution. (**B–D**) Data show the mean ± SEM of the frequency of dividing cells, with each symbol representing the mean of triplicate wells for an individual HD. Data are combined from (**B**) *n* = 4, (**C**) *n* = 2, and (**D**) *n* = 3 independent experiments. (**B** and **D**) Two-tailed paired Student’s *t* test.

**Figure 2:**
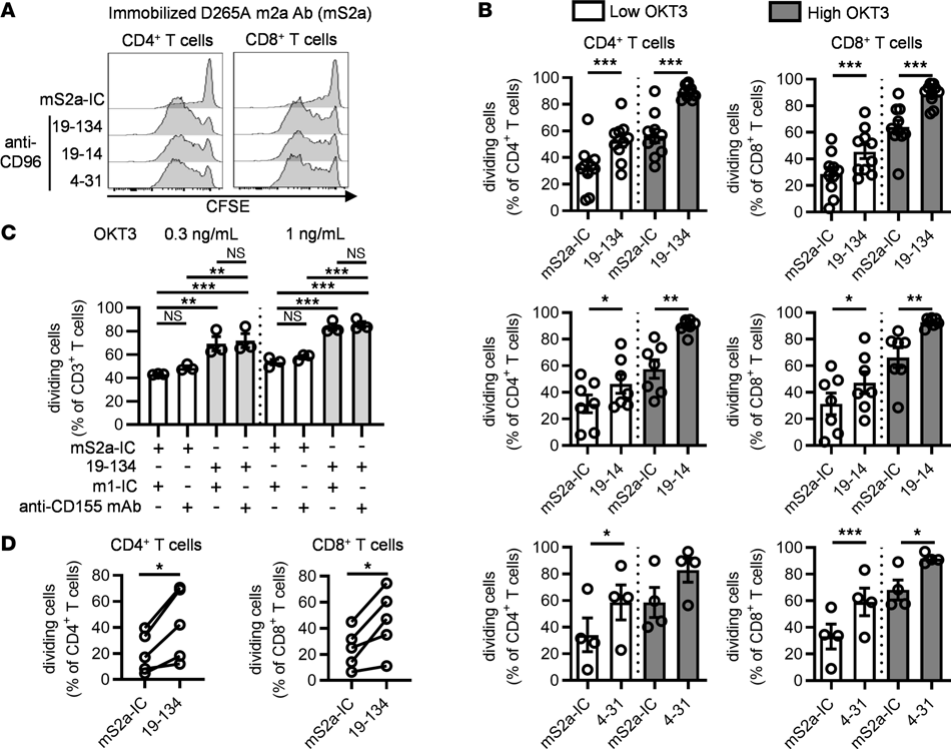
Immobilized CD96 mAbs enhance CD4^+^ and CD8^+^ T cell proliferation. (**A–C**) CFSE-labeled PBMCs were stimulated for 4 days with soluble OKT3 and plate-bound anti-CD96 mS2a antibodies or an isotype control (mS2a-IC), in the presence of (**C**) soluble blocking anti-CD155 mAb or an IC (m1-IC). Cell division among T cell subsets was analyzed by flow cytometry. (**A**) Representative examples of CFSE dilution. (**B** and **C**) Data show the mean ± SEM of the frequency of dividing cells, with each symbol representing the mean of triplicate wells for an individual HD. (**D**) CFSE-labeled CD3^+^ T cells purified from HDs were stimulated for 5 days with soluble anti-CD3/anti-CD28 tetrameric complexes and plate-bound anti-CD96 mS2a antibody (clone 19-134) or an IC. Each data point represents the mean of the frequency of dividing cells from triplicate wells for an individual HD. Data are combined from (**B**) *n* = 6, *n* = 4, and *n* = 2 independent experiments for clones 19-134, 19-14. and 4-31, respectively, and from (**C**) *n* = 2 and (**D**) *n* = 3 independent experiments. *P ≤ 0.05, **P ≤ 0.01, ***P ≤ 0.001. (**B** and **D**) Two-tailed paired Student’s *t* test; (**C**) 1-way ANOVA.

We evaluated 3 different anti-CD96 mAbs that either fully (clones 19-134 and 4-31) or partially (clone 19-14) inhibited the CD155-CD96 interaction (Table 1 and Supplemental Figure 1; supplemental material available online with this article; undefinedDS1) for their ability to promote the proliferation of human T cells. We stimulated CFSE-stained PBMCs isolated from healthy donors with soluble anti-CD3 OKT3 and saturating concentrations (25 μg/ml) of soluble anti-CD96 clone 19-134 and analyzed the frequency of dividing CD4^+^ and CD8^+^ T cells on day 4 after stimulation. Blockade of CD96 was accomplished using murine anti-CD96 IgG2a mAbs bearing a D265A mutation that abolishes binding to FcγRs (21). Under these conditions, resting effector memory and central memory T cells expressed more CD96 compared with naive and terminally differentiated TEMRA cells, and expression was upregulated upon stimulation with soluble OKT3 (Supplemental Figure 2, A and B). Moreover, CD155 was expressed by monocytes at the resting state and was upregulated on a subset of T cells upon activation (refs. 22, 23 and Supplemental Figure 2, C and D). As shown in Figure 1, A and B, clone 19-134 did not significantly alter the proportion of dividing T cells. Similar results were obtained using 2 additional anti-CD96 mAbs (clones 19-14 and 4-31; Figure 1, A, C, and D). Taken together, these data demonstrate that CD96 blockade does not confer a proliferative advantage to anti-CD3–stimulated T cells. Next, we tested whether the activity of anti-CD96 mAbs could be potentiated through antibody immobilization on tissue culture plates, an experimental strategy used for inducing antibody-mediated receptor cross-linking. In contrast with the findings using soluble antibodies, plate-bound anti-CD96 mAbs were able to costimulate the proliferation of CD4^+^ and CD8^+^ T cells (Figure 2, A and B). We also tested whether blocking the CD155-CD96 interaction with an anti-CD155 mAb can affect T cell proliferation. As shown in Figure 2C, the addition of a blocking anti-CD155 mAb failed to enhance T cell proliferation and did not affect the increase in cell proliferation afforded by plate-bound anti-CD96 mAb. As CD96 is expressed by T cells and NK cells in resting human PBMCs (3, 10), we examined whether anti-CD96 mAbs could costimulate purified CD3^+^ T cells. As shown in Figure 2D, immobilized anti-CD96 mAb significantly boosted the proliferation of isolated CD4^+^ and CD8^+^ T cells, demonstrating that anti-CD96 mAbs act directly on T cells. Collectively our data demonstrate that plate-bound anti-CD96 mAbs can directly costimulate T cells, independent of their ability to inhibit the CD96-CD155 interaction.

**Figure 3:**
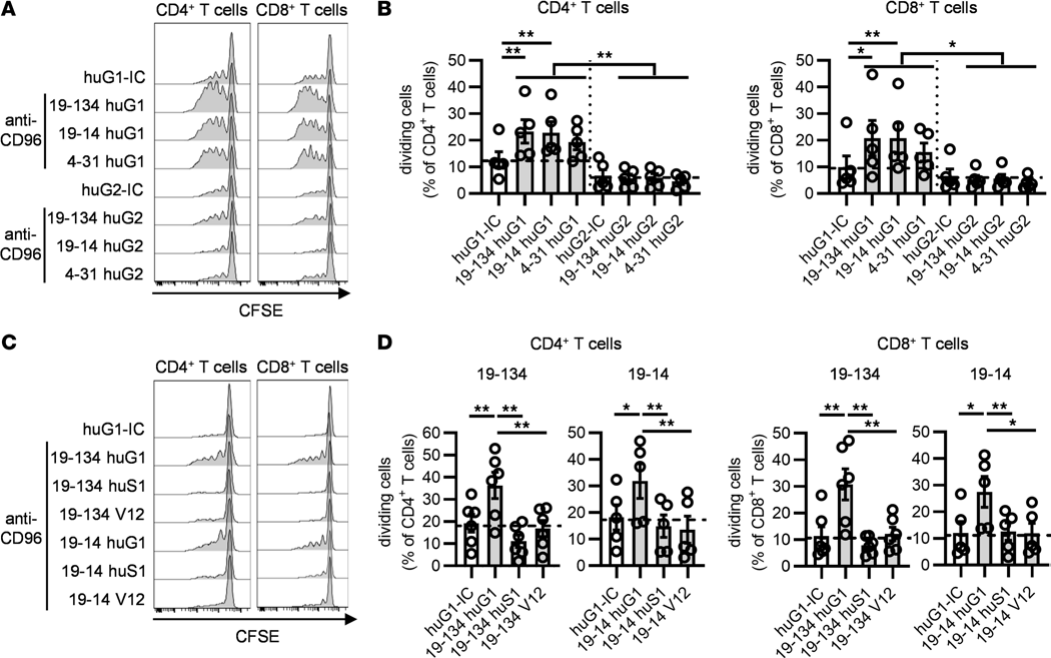
The activity of CD96 mAbs requires FcγR cross-linking. CFSE-labeled PBMCs from HDs were stimulated for 4 days with soluble OKT3 and soluble anti-CD96 mAb variants as indicated, and the proportion of proliferating cells was determined by flow cytometry. (**A** and **B**) The effect of human IgG1 (huG1) and human IgG2 (huG2) anti-CD96 mAbs on CD4^+^ and CD8^+^ T cell proliferation was compared. (**C** and **D**) The effect of huG1, Fc-silent N297S human IgG1 (huS1), and V12 human IgG1 anti-CD96 mAbs on CD4^+^ and CD8^+^ T cell proliferation was compared. (**A** and **C**) Representative examples of CFSE dilution. (**B** and **D**) Data show the mean ± SEM of the frequency of dividing cells, with each symbol representing the mean of triplicate wells for an individual donor and the dotted lines indicating the percentage of dividing cells after stimulation with the isotype controls. Data are combined from (**B**) *n* = 4 independent experiments and (**D**) from *n* = 4 and *n* = 3 independent experiments for clones 19-134 and 19-14, respectively. *P ≤ 0.05, **P ≤ 0.01. One-way ANOVA.

**Figure 4:**
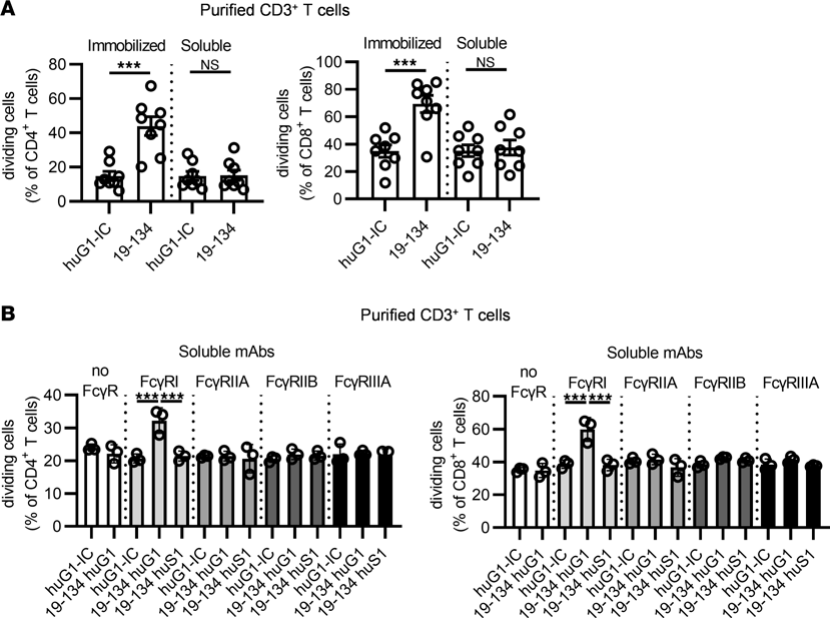
Cross-linking by FcγRI enables the T cell stimulatory property of soluble anti-CD96 huG1 mAb. CFSE-labeled purified CD3^+^ T cells from HDs were stimulated for 4 days, and cell division was analyzed by flow cytometry. (**A**) T cells were stimulated with plate-bound OKT3 and either plate-bound or soluble anti-CD96 huG1 antibody (clone 19-134) or an IC. (**B**) T cells were stimulated with plate-bound OKT3 and soluble huG1, Fc-silent N297S huG1 (huS1) 19-134 antibody, or an IC, in the presence of plate-bound individual recombinant FcγR. Data show (**A**) the mean ± SEM of the frequency of dividing cells, with each symbol representing the mean of triplicate wells for an individual HD, and (**B**) the mean ± SEM of the frequency of dividing cells in triplicate wells from 1 HD (representative of 4), with each symbol representing data from an individual well. Data are from (**A**) *n* = 4 and (**B**) *n* = 2 independent experiments. ***P ≤ 0.001. One-way ANOVA.

To investigate if FcγR engagement can substitute for the requirement for mAb immobilization, we isotype-switched FcγR-disabled anti-CD96 mouse IgG2a (D265A) mAbs to FcγR-competent human IgG1 and IgG2 isotypes. While human IgG1 exhibits binding to all FcγRs, human IgG2 binds to FcγRIIA and FcγRIIIA, albeit with a lower affinity than IgG1 (24). For each antibody clone, we confirmed that the 2 isotypes displayed equivalent binding capacities to CD96, as demonstrated by their similar EC_50_ values (Table 2). Remarkably, soluble human IgG1, but not human IgG2 variants, augmented CD4^+^ and CD8^+^ T cell division in the PBMC proliferation assay, suggesting that the stronger and broader FcγR binding activity of IgG1 was required for the observed costimulatory effects (Figure 3, A and B). To confirm that the costimulatory effects of the anti-CD96 IgG1 mAbs were dependent on coengagement of FcγRs, we produced FcγR-silent human IgG1 versions (N297S; refs. 25, 26) of anti-CD96 clones 19-134 and 19-14, which had the most potent effect on T cell proliferation. Table 2 shows that antibody binding to CD96 was not affected by the N297S mutation. Increased proliferation of CD4^+^ and CD8^+^ T cells elicited by soluble anti-CD96 IgG1 clones was completely abolished by the N297S mutation, demonstrating that coengagement of FcγRs is essential for their costimulatory effects (Figure 3, C and D). To gain a better understanding of which FcγR was required in mediating the activity of the anti-CD96 IgG1 mAbs, we generated a mutant (IgG1 V12) that possesses significantly reduced affinity to FcγRI, FcγRIIA^H131^, and FcγRIIIA but stronger binding to FcγRIIB (27). We evaluated 2 anti-CD96 clones (19-134 and 19-14) in the IgG1 V12 format, but neither mAb was active (Figure 3, C and D), corroborating the hypothesis that the higher affinity of IgG1 for FcγRI, FcγRIIA, and FcγRIIIA was essential for antibody-mediated CD96 cross-linking and subsequent T cell costimulation. To address the source of FcγRs in the PBMC proliferation assay, we analyzed the expression of FcγRI, FcγRIIA/B, and FcγRIIIA on various leukocytes from PBMCs. FcγRI, FcγRIIA/B, and FcγRIIIA were expressed on monocytes, B cells, and NK cells (Supplemental Figure 3) in the expected pattern (28). In contrast, neither resting nor OKT3-activated T cells expressed these FcγRs (Supplemental Figure 3), indicating that anti-CD96 mAb cross-linking was mediated through a *trans-*interaction with FcγRs expressed on accessory cells. Consistent with this notion, soluble anti-CD96 (human IgG1) did not stimulate highly purified CD3^+^ T cells, unlike plate-bound mAb (Figure 4A). To investigate which FcγR is required for mediating the effects of anti-CD96 mAb, we employed individual recombinant FcγRs coated onto plastic, together with highly purified CFSE-labeled CD3^+^ T cells, and showed that FcγRI was uniquely able to restore the activity of soluble anti-CD96 human IgG1 (Figure 4B).

Collectively, our data demonstrate that soluble anti-CD96 mAbs of the IgG1 subclass enhance the proliferation of CD4^+^ and CD8^+^ T cells, dependent on mAb cross-linking through Fc domain *trans*-interaction with FcγRI.

### Agonistic anti-CD96 mAb counters suppression by Tregs.

**Figure 5:**
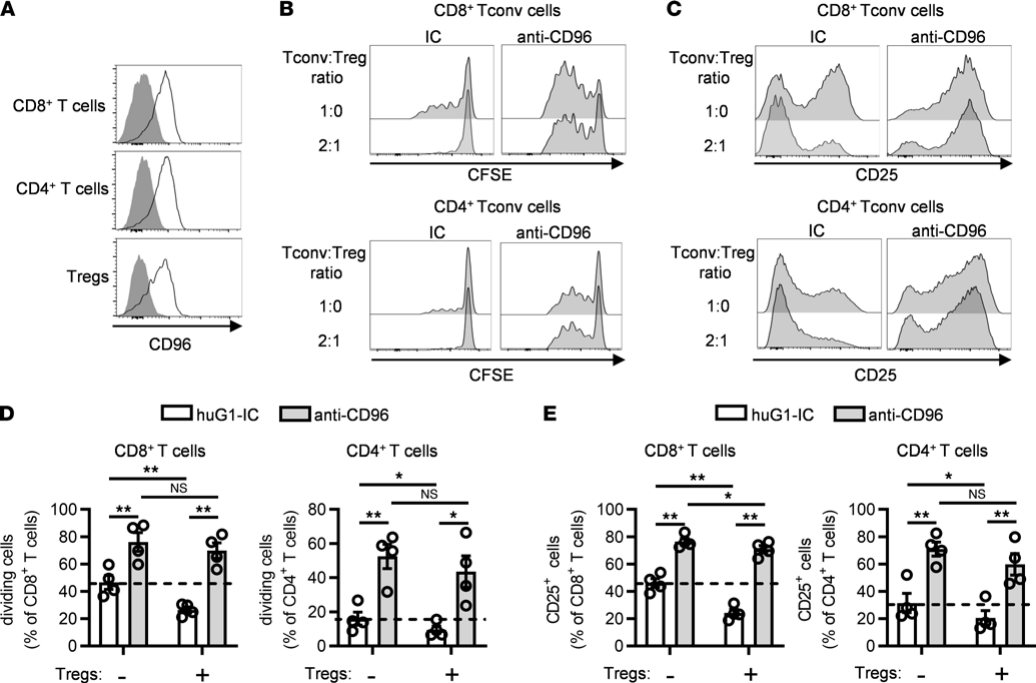
Antibody targeting of human CD96 can overcome Treg suppression. (**A**) The expression of CD96 on CD8^+^ T cells, conventional CD4^+^ T cells (CD25^lo^ CD127^hi^), and Tregs (CD25^hi^ CD127^lo^) was analyzed by flow cytometry in PBMCs from HDs. Data show 1 representative example of 8 HDs. (**B–E**) CFSE-stained Treg-depleted CD3^+^ T (Tconv) cells were stimulated for 4 days with plate-bound OKT3 and plate-bound anti-CD96 huG1 antibody (clone 19-134) or a matching IC, either alone or in the presence of purified unlabeled Tregs. (**B** and **C**) Representative examples of (**B**) CFSE dilution and (**C**) CD25 expression in CFSE^+^ CD8^+^ and CFSE^+^ CD4^+^ Tconv cells. (**D** and **E**) Data show the mean ± SEM of the frequency of (**D**) dividing cells and (**E**) CD25^+^ cells in the CFSE^+^CD4^+^ and the CFSE^+^CD8^+^ Tconv cell populations, with each symbol representing the mean of triplicate wells for an individual HD. Data are combined from 2 independent experiments. *P ≤ 0.05, **P ≤ 0.01. Two-way ANOVA.

Tregs exert a dominant role in maintaining self-tolerance and suppressing antitumor T cell responses (29), but the role of CD96 on Tregs is currently unknown. Flow cytometric analysis revealed that peripheral blood Tregs expressed CD96 similarly to conventional CD4^+^ and CD8^+^ T cells (Figure 5A). To assess if the presence of increasing numbers of Tregs would negate the costimulatory effect of anti-CD96 mAbs, highly purified, CFSE-labeled CD3^+^CD25^–^CD127^+^ (98.1% ± 0.5%) conventional/effector T (Tconv) cells were stimulated with anti-CD3 and either anti-CD96 or an isotype-matched control antibody. In some cultures, purified unlabeled CD4^+^CD25^+^CD127^–^ Tregs (93.6% ± 1.8% purity) were added to obtain a Tconv cell/Treg ratio of 2:1 or 3:1. Tconv cell proliferation and activation were determined by measurement of CFSE dilution and upregulation of CD25, respectively, after 4 days. As expected, the addition of purified Tregs suppressed the proliferation of CD4^+^ and CD8^+^ Tconv cells and reduced expression of CD25 (Figure 5, B–E). However, when anti-CD96 mAb was present, both Tconv cell proliferation and CD25 expression were restored to levels seen in the absence of Tregs (Figure 5, B–E). These data support the notion that costimulation of Tconv cells by anti-CD96 mAb overcomes, to a large extent, the suppression exerted by Tregs.

### Gene expression profiling reveals augmentation of multiple T cell activation pathways by CD96.

**Figure 6:**
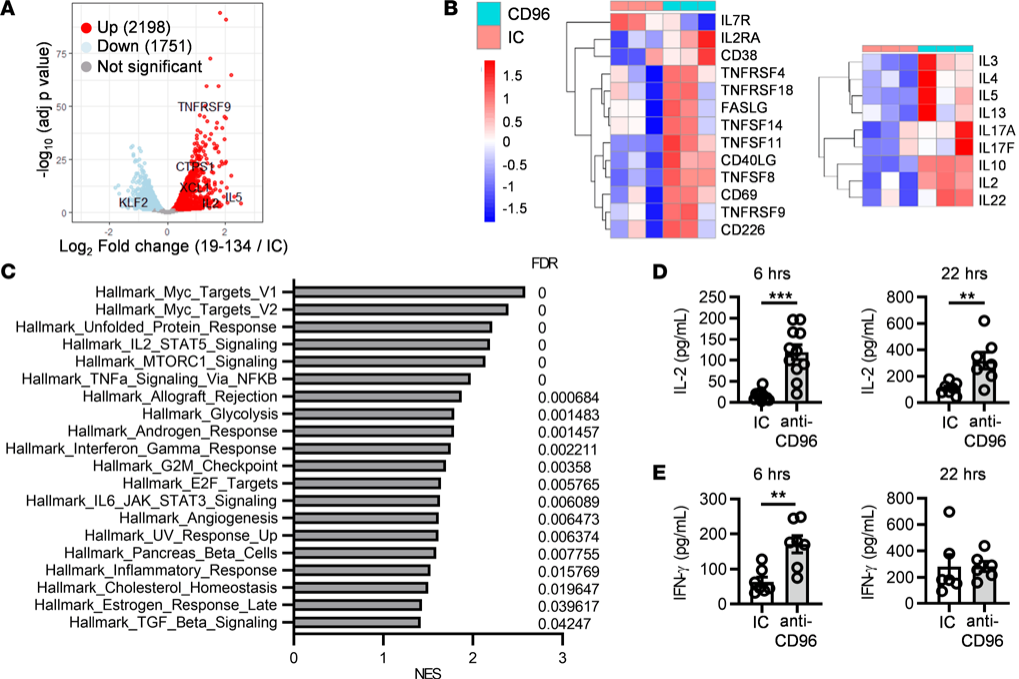
Anti-CD96 mAb triggers a transcriptional program associated with T cell proliferation and effector function. Purified CD3^+^ T cells from 3 HDs were stimulated for 6 or 22 hours with plate-bound OKT3 and huG1 anti-CD96 mAb (clone 19-134) or an IC. (**A–C**) RNA-Seq analysis was performed after 6-hour stimulation; differential gene expression was determined with DESeq2 package. (**A**) Volcano plot representing differentially expressed genes. (**B**) Heatmaps plotted as regularized log-transformed expression values, illustrating differential gene expression of selected activation markers (left) and cytokines (right). (**C**) Gene set enrichment analysis showing hallmark gene sets significantly enriched in the anti-CD96 group versus the IC group. NES, normalized enrichment score. (**D**) IL-2 and (**E**) IFN-γ were quantified by ELISA in culture supernatants harvested after 6 and 22 hours. Data show the mean ± SEM, with each data point representing the mean of triplicate wells for an individual donor. Data are combined from (**D**) *n* = 6 and *n* = 4 independent experiments for 6 and 22 hours, respectively, and (**E**) from *n* = 4 independent experiments. **P ≤ 0.01, *** *P* ≤ 0.001. Two-tailed paired Student’s *t* test.

To gain further insights into the downstream events triggered by anti-CD96 mAbs, we performed RNA-Seq on T cells from 3 healthy donors that were stimulated for 6 hours with anti-CD3 together with either anti-CD96 or a matching isotype control mAb. Differential gene expression analysis showed that 2,198 gene transcripts were significantly upregulated and 1,751 gene transcripts were significantly downregulated by the anti-CD96 mAb (adjusted *P* ≤ 0.05), indicating that CD96 engagement results in early transcriptional changes in activated T cells (Figure 6A). Gene transcripts typically modulated upon T cell activation were affected by anti-CD96 treatment. Thus, while *IL7R* was downregulated, *CD69*, *CD25*, *CD38*, *FASL*, and *CD226*, as well as multiple members of the TNF and TNFR superfamilies, such as *CD40L*, *TNFSF14* (also known as LIGHT), *TNFRSF9* (also known as 4-1BB), *TNFRSF4* (also known as OX40), and *TNFRSF18* (also known as GITR), were upregulated (Figure 6B, left). Furthermore, anti-CD96 treatment increased the expression of multiple cytokines, including *IL2*, *IL4*, *IL5*, *IL13*, *IL17A*, *IL17F*, *IL10*, and *IL22*, consistent with an augmentation of T cell effector function by CD96 (Figure 6B, right). We conducted gene set enrichment analysis using the hallmarks gene sets from the Molecular Signatures Database (30) and pathway analysis using Ingenuity Pathway Analysis (IPA). Multiple hallmarks associated with an activated T cell signature were significantly enriched in the anti-CD96 treatment group. These included gene signatures associated with cell cycle progression, such as Myc, e2F, or G_2_/M checkpoint, consistent with the observed increase in T cell proliferation upon anti-CD96 treatment (Figure 6C). Signatures related to metabolic reprogramming (“MTORC1 signaling” and “Glycolysis”), effector differentiation (“IFN-γ response”), as well as sustained proliferation and survival (“IL-2/STAT5 signaling” and “TNF-α signaling via NF-κB”) were also enriched in the anti-CD96 treatment group. Consistently, the hallmark of the unfolded protein response, which is known to contribute to the regulation of T cell proliferation and effector function (31), was significantly upregulated following anti-CD96 treatment (Figure 6C). Quantification of cytokine production in the supernatant of T cells stimulated for 6 or 22 hours showed that anti-CD96 significantly upregulated IL-2 production by CD3^+^ T cells at both time points, while IFN-γ production was augmented at 6 hours (Figure 6, D and E). Hence, increased gene transcription correlated with elevated protein levels for IL-2 and IFN-γ. Moreover, we showed that agonist anti-CD96 mAb provided direct costimulation to CD4^+^ and CD8^+^ isolated T cells, resulting in enhanced IL-2 production from each of these cell types in addition to promoting independent signals for CD4^+^ and CD8^+^ T cell proliferation (Supplemental Figure 4).

Furthermore, IPA identified a broad range of upstream regulators predicted to be activated and a smaller number of regulators predicted to be inhibited by CD96 stimulation (Supplemental Figure 5, A and B). TCR, CD3, and CD28 were highlighted as potential positive upstream regulators of the gene signature induced by anti-CD96 mAb, suggesting that CD96 engagement elicits signaling pathways that overlap and strengthen those emanating from the engagement of the TCR and CD28 (Supplemental Figure 5A). In agreement with this, transcription factors and signaling kinases triggered by the integrated response to TCR and CD28 engagement, such as Myc, Jun, NF-κB, Mek/MAP2K1/2, PI3K/Akt, and p38 MAPK, were additionally identified as upstream activating regulators (Supplemental Figure 5A).

Collectively our transcriptomic data indicated that CD96 engagement triggers multiple signaling pathways associated with increased T cell proliferation and effector function and identified several candidate molecules that could mediate signaling downstream of CD96.

### Agonist anti-CD96 mAb augments the proliferation of TILs.

**Figure 7:**
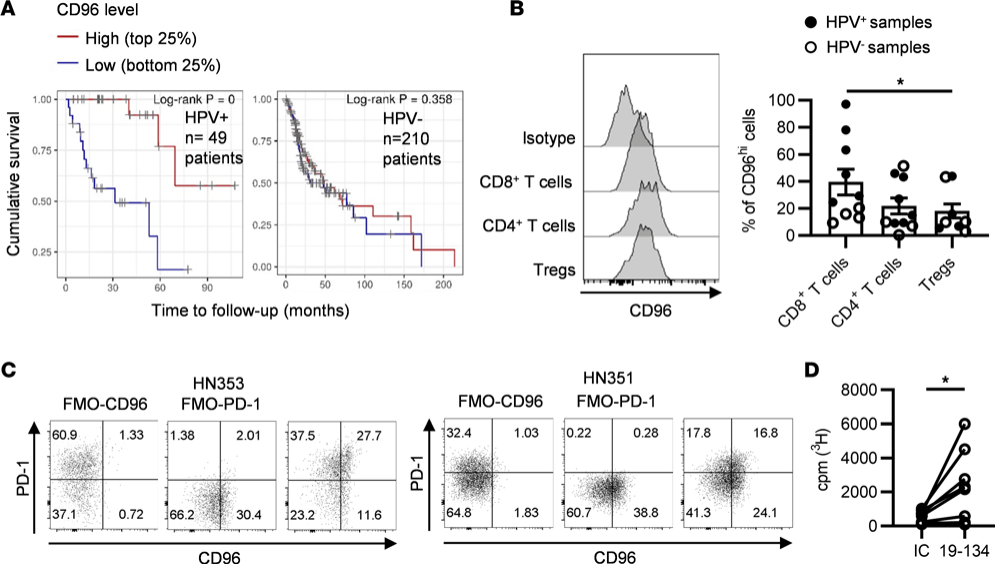
Anti-CD96 mAb stimulates the proliferation of tumor-infiltrating lymphocytes from head and neck squamous cell carcinomas. (**A**) Using data from The Cancer Genome Atlas, Kaplan-Meier curves of cumulative survival were generated for HPV^+^ and HPV^–^ patients with head and neck squamous cell carcinoma (HNSCC), based on CD96 expression levels. (**B**) The expression of CD96 on tumor-infiltrating CD8^+^ T cells, conventional CD4^+^ T cells (CD4^+^Foxp3^–^), and Tregs (CD4^+^Foxp3^+^) isolated from *n* = 10 HNSCC biopsies was analyzed by flow cytometry. Data show (left) representative histogram overlays of CD96 expression and (right) the frequency of CD96^hi^ cells in each T cell subset. (**C**) Coexpression of CD96 and PD-1 on tumor-infiltrating CD8^+^ T cells from *n* = 11 HNSCC biopsies was analyzed by flow cytometry. Data show dot plots of marker coexpression. FMO, fluorescence minus one. (**D**) Tumor-infiltrating T cells from *n* = 13 HPV^+^ tumors were stimulated with plate-bound OKT3 and anti-CD96 huG1 mAb (clone 19-134) or a matching isotype control for 5 days. Cells were pulsed with tritiated thymidine in the last 16 hours of culture. Each symbol represents the mean of the counts per minute of triplicate wells for each individual patient. Data are combined from *n* = 12 independent experiments. *P ≤ 0.05. (**B**) Friedman’s test and Dunn’s multiple comparisons test; (**D**) 2-tailed Wilcoxon’s paired test.

Given that anti-CD96 mAbs were able to costimulate peripheral blood T cells, we asked if this approach could also promote the proliferation of TILs, which are known to exist in various dysfunctional states (32). Using publicly available data from The Cancer Genome Atlas database through the Tumor Immune Estimation Resource (33), we first investigated the effect of CD96 expression on the survival of patients with HPV^+^ or HPV^–^ head and neck squamous cell carcinoma (HNSCC). Interestingly, high CD96 transcript levels correlated with improved survival in HPV^+^ patients with HNSCC (Figure 7A, left). In contrast, CD96 expression was not associated with better survival in HPV^–^ patients with HNSCC (Figure 7A, right), which typically display limited T cell infiltration and worse clinical outcome (34, 35). Next, we used flow cytometry to examine CD96 expression on T cell subsets isolated from fresh HNSCC tumor biopsies (patient characteristics are included in Table 3). CD96 was expressed on CD8^+^ T cells, CD4^+^Foxp3^–^ Tconv cells, and CD4^+^ Foxp3^+^ Tregs (Figure 7B). Although ranging widely between patients, on average expression of CD96 on CD8^+^ T cells was higher than that seen on the other T cell subsets analyzed (Figure 7B). Furthermore, we evaluated whether CD96 is coexpressed with the inhibitory receptor PD-1, typically found on chronically stimulated and/or exhausted tumor-infiltrating CD8^+^ T cells (36). Figure 7C shows that PD-1 expression on CD8^+^ T cells from HNSCC tumors varied among patients, and expression of CD96 could be detected on a substantial proportion of the PD-1–bright and PD-1–dim T cells (Figure 7C).

To test whether anti-CD96 mAbs are capable of costimulating TILs, we isolated lymphocytes from HPV^+^ HNSCC tumors and measured T cell proliferation in response to plate-bound anti-CD3 and anti-CD96. On average, the percentages of tumoral CD8^+^ T cells, CD4^+^ Tconv cells, and CD4^+^ Tregs of the CD3^+^ T cells were 35.6% ± 5.2%, 42.7% ± 5.9%, and 15.9% ± 2.2%, respectively. The data presented in Figure 7D show that TILs proliferated more extensively when cultured with anti-CD3 and anti-CD96 mAb compared with incubation with anti-CD3 and a control mAb, highlighting CD96 as a potential target to reinvigorate anticancer T cells.

## Discussion

Despite the success of targeting the PD-1/PD-L1 inhibitory axis, there remains a strong incentive to discover additional immunomodulatory targets, driven primarily by the need to extend the response rate and durability offered by current treatments. Herein, we provide data that suggest that mAbs targeting human CD96, a member of IgSF, expressed at low levels on naive T cells but strongly upregulated during T cell activation, are potent stimulators of T cell activation and proliferation. Although earlier studies, which primarily focused on murine NK cell responses, suggested that CD96 could function as an inhibitory receptor (14, 15), our data using human T cells do not support this notion. Instead, we provide evidence that CD96 is a bona fide costimulatory receptor for human T cells. First, we showed that soluble Fc-silent mAbs that block the interaction of CD96 with its ligand CD155 did not exert functional effects (Figure 1), whereas the same mAbs were stimulatory when coated on tissue culture plastic (Figure 2). Second, the conversion of Fc-silent anti-CD96 mAbs to Fc-competent mAbs of the IgG1 subclass endowed them with the capacity to costimulate T cells without the need for coating (Figure 3). Third, we demonstrated that the T cell costimulatory effects of soluble anti-CD96 IgG1 are critically dependent on cross-linking mediated through *trans*-binding to FcγRI (Figures 3 and 4). We interpret these results as evidence that immobilization of anti-CD96 mAbs either by coating on synthetic surfaces or more physiologically through coengagement of FcγRI results in CD96 clustering on the T cell surface, which subsequently leads to stimulation of intracellular signaling. Our findings are consistent with those of a recent study demonstrating that coupling of anti-CD96 mAbs to beads provided a costimulatory signal to T cells (17). Our data extend previous findings by demonstrating the importance of the antibody Fc domain in driving the functional activity of anti-CD96 mAbs. These findings should, therefore, guide future development of agonist anti-CD96 mAb aimed at enhancing suboptimal antitumor responses. In this context, it is well-known that antitumor T cell responses are hindered by Tregs, and, therefore, our data showing that anti-CD96 mAb was highly effective in overcoming suppression by Tregs is noteworthy (Figure 5). Therefore, we anticipate that anti-CD96 mAbs remain capable of augmenting Tconv cell responses, in spite of the presence of increasing numbers of Tregs within the tumor microenvironment.

Mechanistically, CD96 costimulation could lessen Treg-mediated suppression in a number of ways. First, by augmenting IL-2 secretion (Figure 6 and Supplemental Figure 4) and the expression of CD25 on CD4^+^ and CD8^+^ Tconv cells (Figure 5), the ability of Tregs to deprive responder T cells of IL-2 (29) is likely to be reduced, thus increasing the bioavailability of IL-2 to Tconv cells. Second, our transcriptomic data and pathway analysis suggested convergence of CD96 signaling pathways with those downstream of CD3 and CD28 (Figure 6 and Supplemental Figure 5). This is predicted to reduce the dependency of Tconv cells on costimulation via CD80/86 and CD28 and, therefore, could circumvent Treg-mediated suppression exerted by CTLA-4–expressing Tregs (29). Third, our transcriptomic analysis also showed that CD96 costimulation upregulated several costimulatory receptors and ligands, including OX40, GITR, 4-1BB, CD40 ligand, and CD226, which could further lower the activation threshold of Tconv cells and impede Treg suppression. Although our data offer plausible mechanisms of how Tconv cells resist suppression, an alternative hypothesis might be that anti-CD96 antibodies modulate Tregs directly, as these cells also express CD96, a possibility that will be examined in future studies.

From the perspective of developing new anticancer immunotherapies, the finding that CD96 costimulation is able to augment the proliferation of intratumoral T cells from HPV^+^ HNSCC is particularly encouraging. A recent study showed that intratumoral HPV-specific PD-1^+^ CD8^+^ T cells can be distinguished by expression of TCF-1 and TIM-3, markers that are used to identify stem cell–like and terminally differentiated T cells, respectively (36). Interestingly, the authors of that study demonstrated that it is the stem cell–like CD8^+^ T cells that proliferate extensively upon in vitro stimulation with the cognate HPV peptide (36). Herein, we showed that CD96 expression on tumor-infiltrating CD8^+^ T cells is heterogeneous, being expressed on a fraction of PD-1–bright as well as on PD-1–dim T cells. Therefore, it would be interesting to dissect the role of CD96 further by examining how CD96 costimulation affects different HPV-specific CD8^+^ T cell subsets. Such studies will inform of more effective strategies to reinvigorate anticancer T cells in patients.

## Methods

### Healthy donors and PBMC preparation.

Anonymized leukocyte cones were obtained from the National Blood Service (Southampton, United Kingdom). PBMCs were isolated by density gradient centrifugation using Lymphoprep (Stemcell) according to the manufacturer’s instructions.

### Patient with HNSCC and TIL preparation.

Tumor biopsies were obtained from patients with HPV^+^ and HPV^–^ tumors at Poole Hospital NHS Foundation Trust (United Kingdom). TILs were isolated from freshly resected tumor tissues using enzymatic and mechanical dissociation. Briefly, tumor samples were cut into small fragments and incubated at 37°C for 20 minutes under agitation in RPMI containing 0.15 WU/mL of liberase DL (Roche) and 800 units/mL DNase I (MilliporeSigma). RPMI containing 10% fetal calf serum was added to the digested fragments, and samples were then dispersed through a 100 μm strainer, centrifuged, and washed once before phenotyping or proliferation studies.

### Immunomagnetic selection.

CD3^+^ T cells were isolated from PBMCs using the EasySep human T cell negative selection kit (Stemcell). CD4^+^CD25^hi^CD127^lo^ Tregs and CD3^+^CD25^lo^CD127^hi^ conventional/effector cells were purified from the CD3^+^ T cell fraction using the EasySep human CD4^+^CD127^lo^CD25^+^ regulatory T cell isolation kit (Stemcell). CD8^+^ T cells were purified from the CD3^+^ T cell fraction using the EasySep human CD8 Positive Selection kit II (Stemcell), and the CD8^–^ cell fraction was used as the CD4^+^ T cell fraction.

### Antibodies for functional assays.

The OKT3 hybridoma was obtained from the ATCC, and OKT3 mIgG2a antibody was purified from hybridoma tissue culture supernatant by affinity chromatography. Antibodies against human CD96 were generated by BliNK Biomedical. Briefly, mice were immunized with plasmid DNA–encoding human CD96v2 and boosted with CD96v2-expressing CHO cells. Spleen and lymph nodes of immunized animals were harvested, and individual cells secreting anti-CD96 mAbs were identified using the ImmunoSpot Array Assay on Chip technology. mRNA was isolated from single cells and cDNA sequences encoding the variable regions of the heavy (VH) and light (VL) chains of IgG were amplified by RT-PCR. VH and VL sequences were cloned into an expression vector encoding a D265A mouse IgG2a backbone that was used to transfect CHO-K1 cells (ATCC). Binding of recombinant mAbs to human CD96 was confirmed by ELISA and surface plasmon resonance (Biacore) using recombinant human CD96-Fc protein produced in-house. Recognition of native CD96 by CD96 mAbs was confirmed by flow cytometry using CD96v2-expressing CHO cells and PBMCs. To determine EC_50_ values, CD96v2-expressing CHO cells were incubated at 4°C for 30 minutes with titrated amounts of each anti-CD96 clone or an isotype control and washed twice, and mAb binding was detected using a fluorescently labeled secondary antibody and flow cytometry. To assess the ability of anti-CD96 mAbs to inhibit the interaction between CD155 and CD96 and determine IC_50_ values, CD96-expressing CHO cells were incubated with titrated amounts of anti-CD96 mAbs and biotinylated human CD155-Fc produced in-house for 30 minutes at 4°C. Cells were washed and CD155-Fc binding was detected using FITC-conjugated streptavidin (BD Biosciences) and flow cytometry analysis. Dose-response curves were obtained from GraphPad Prism software, using a nonlinear regression fit with a variable slope model, and used to determine the IC_50_. Antibody VH and VL sequences were cloned into human IgG1 (huG1), N297S human IgG1 (huS1), V12 human IgG1, or human IgG2 (huG2) backbones (MImAbs), and the EC_50_ of the various variants was determined as described above.

### Proliferation and Treg suppression assays.

All cell cultures were performed at 37°C in 5% CO_2_ using RPMI supplemented with 10% heat-inactivated fetal calf serum (MilliporeSigma), 2 mM glutamine (Thermo Fisher Scientific), 1 mM pyruvate (Thermo Fisher Scientific), and 100 IU/mL penicillin and 100 μg/mL streptomycin (MilliporeSigma). When required, antibodies or recombinant proteins diluted in bicarbonate coating buffer (50 mM, pH 9.6) were immobilized on tissue culture plates for a minimum of 3 hours, using 100 μL per well, and plates were washed 3 times with PBS before addition of cells. A concentration of 500 ng/mL OKT3, 10 μg/mL D265A mouse IgG2a antibodies, 2.5 μg/mL human IgG1 variants, and 2.5 μg/mL recombinant FcγRI, FcγRIIA, FcγRIIB, and FcγRIIIA (R&D Systems) was used for plate coating. For CD155-CD96-blocking experiments, 25 μg/mL of soluble D265A mouse IgG2a antibodies was used. For human IgG1, human IgG2, V12 human IgG1, and N297S human IgG1 variant comparison analysis, 1 μg/mL soluble mAb was used. When indicated, 25 μg/mL anti-CD155 mouse IgG1 (m1) antibody (clone SKII.4, Biolegend) or m1 isotype control (MOPC-21, BioXCell) was added to the cultures.

To assess T cell division, PBMCs or purified T cell subsets isolated from healthy donors were stained with 1.5 μM CFSE (Thermo Fisher Scientific). For proliferation assays, cells were distributed in triplicates in U-bottom 96-well plates, at 10^5^ cells per well, except in the assays using recombinant FcγRs, where 7 × 10^4^ T cells per well were used. PBMCs were stimulated with 0.1–1 ng/mL soluble OKT3, while purified T cell subsets were stimulated with soluble anti-CD3/anti-CD28 tetrameric complexes (Immunocult, Stemcell) or plate-bound OKT3, as indicated. Soluble or plate-bound anti-CD96 mAbs were added to the cultures, as specified in the text. For Treg assays, CFSE-stained Tconv cells were distributed at 7.5 × 10^4^ cells per well in triplicates in U-bottom 96-well plates coated with OKT3 and anti-CD96 mAbs or an isotype control and cocultured with unstained Tregs at a 2:1 or 3:1 Tconv cell/Treg ratio. CFSE dilution was analyzed by flow cytometry on day 4 or 5 after stimulation, as indicated.

For TIL proliferation assays, freshly isolated cells were distributed at 7.5 × 10^4^ cells per well in triplicates in U-bottom 96-well plates coated with OKT3 and anti-CD96 huG1 mAbs or an isotype control. Four days after stimulation, 1 μCi per well of tritiated thymidine was added, and cells were harvested after a further 16-hour culture.

### Flow cytometry.

Antibodies against human CD3 (BW264/56) and CD45RA (T6D11) were purchased from Miltenyi Biotec. Antibodies against human CD3 (UCHT1), CD25 (BC96), CD127 (A019D5), PD-1 (EH12.2H7), CCR7 (G043H7), and CD155 (SKII.4) were obtained from Biolegend. Fixable viability dye and antibodies against human CD4 (RPA-T4), CD8α (RPA-T8), and Foxp3 (PCH101) were purchased from Thermo Fisher Scientific, and antibody against human CD96v2 (628211) was obtained from R&D Systems. Fluorescently labeled F(ab′)2 against FcγRI (10.1), FcγRIIA/B (AT10), and FcγRIIIA (3G8) were provided by Mark Cragg, University of Southampton. FcγR staining was performed in PBS/1% BSA, without a prior FcγR blocking step. For CFSE proliferation assays and analysis of CD96 and CD155 expression, cells were incubated for 10 minutes at 4°C with 10% heat-inactivated AB serum (MilliporeSigma) prior to surface staining. For TIL phenotyping, cells were incubated with human FcR blocking reagent (Miltenyi Biotec) for 10 minutes at 4°C prior to surface staining. When required, intracellular staining was performed using the Foxp3 staining buffer kit (Thermo Fisher Scientific). Samples were analyzed with a FACSCanto II flow cytometer and DIVA Software (BD Biosciences), FCS Express (De Novo Software), or FlowJo software (version 10).

### IL-2 and IFN-γ ELISA.

Purified T cells were stimulated with immobilized OKT3 (500 ng/mL) and immobilized anti-CD96 huG1 mAb (2.5 μg/mL, clone 19-134) or an isotype control, and supernatants were harvested after 6 hours. The following capture/detection antibody pairs were purchased from Biolegend: MQ1-17H12/Poly5176 and MD-1/4S.B3 for IL-2 and IFN-γ ELISAs, respectively. Maxisorp plates (Nunc) were coated overnight with capture antibodies, plates were blocked with 1% BSA/PBS for an hour, and samples were incubated at room temperature for 90 minutes. For detection, high-sensitivity HRP-linked streptavidin (Pierce) and OPD substrate (MilliporeSigma) were used.

### Transcriptomics.

Purified T cells were stimulated for 6 hours with immobilized OKT3 (500 ng/mL) and immobilized anti-CD96 huG1 mAb (2.5 μg/mL, clone 19-134). Cells were washed, and RNA was isolated using the Qiagen RNeasy Plus Mini kit. The purity, concentration, and integrity of the RNA were assessed using a Nanodrop Spectrophotometer (Thermo Fisher Scientific) and an Agilent 2100 Bioanalyzer (Agilent Technologies). Directional paired-end libraries (150 bp) were sequenced on an Illumina NovaSeq 6000 S2 system (Illumina) at Eurofins Genomics. Quality control analysis of the RNA-Seq data was conducted using FastQC. Sequencing reads were mapped to the hg38 human reference genome using STAR (37) and counted using FeatureCount (38). To identify differentially expressed genes, the R package DESeq2 (version 1.26.0) was used with the design formula “~donor + condition.” Genes with a FDR of equal to or less than 0.05 were considered as significantly different. To generate a matrix of regularized counts for sample visualizations, DESeq2 rlog transformation of the count data was used to stabilize the variance across the mean. To identify functional categories, genes were ranked according to log-fold change (LFC), using apeglm to model the distribution of LFCs (39). The GSEA_v4.0.3 software (UCSD and Broad Institute, ref. 40) was used to calculate normalized enrichment scores and FDR values for the 50 Hallmark gene sets. Subsequent data analysis used IPA (Qiagen). The RNA-Seq data are available from the NCBI Gene Expression Omnibus (GEO) database (GSE193864).

### Survival plots generation for patients with HNSCC.

The Tumor Immune Estimation Resource (33) was used to generate Kaplan-Meier survival curves for HPV^+^ and HPV^–^ patients with HNSCC, based on publicly available data from The Cancer Genome Atlas database. Patients were dichotomized based on CD96 expression levels and log-rank tests were used to compare survival curves and determine *P* values.

### Statistics.

Statistical analysis was performed using GraphPad Prism (v8.2.1) software. Statistical tests applied are indicated throughout and include the log-rank test, 2-tailed paired Student’s *t* test, 1-way ANOVA, 2-way ANOVA, Friedman’s test, Dunn’s multiple comparisons test, and 2-tailed Wilcoxon’s paired test. *P* values of less than 0.05 were considered significant.

### Study approval.

Use of human healthy donor samples was approved by the University of Southampton local ethical committee (ERGO II 19660.A4) and was in accordance with the Declaration of Helsinki. The study of HNSCC samples was approved by the Medical Research and Ethics Committee (South Central-Hampshire B, Bristol, United Kingdom) (MREC 09/H0501/90), and written informed consent was obtained from all patients.

## Author contributions

AR and AAS designed the study. AR, FMI, CNB, SLB, and FRD acquired data. AR, FMI, SMT, CNB, XP, and AAS analyzed and/or interpreted data. EVK provided patients samples. AR, SMT, and AAS wrote the manuscript.

## Supplementary Material

Supplemental data

## Figures and Tables

**Table 1:**
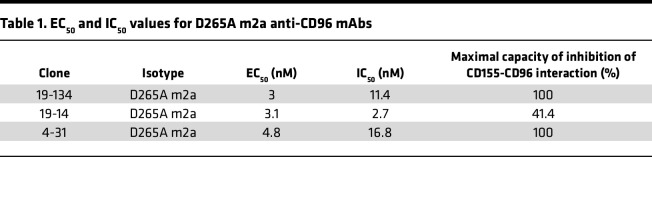
EC_50_ and IC_50_ values for D265A m2a anti-CD96 mAbs

**Table 2:**
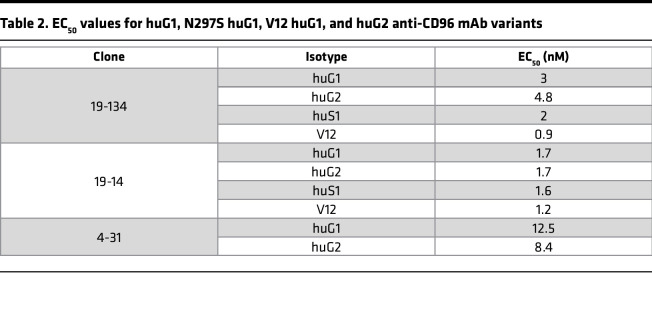
EC_50_ values for huG1, N297S huG1, V12 huG1, and huG2 anti-CD96 mAb variants

**Table 3:**
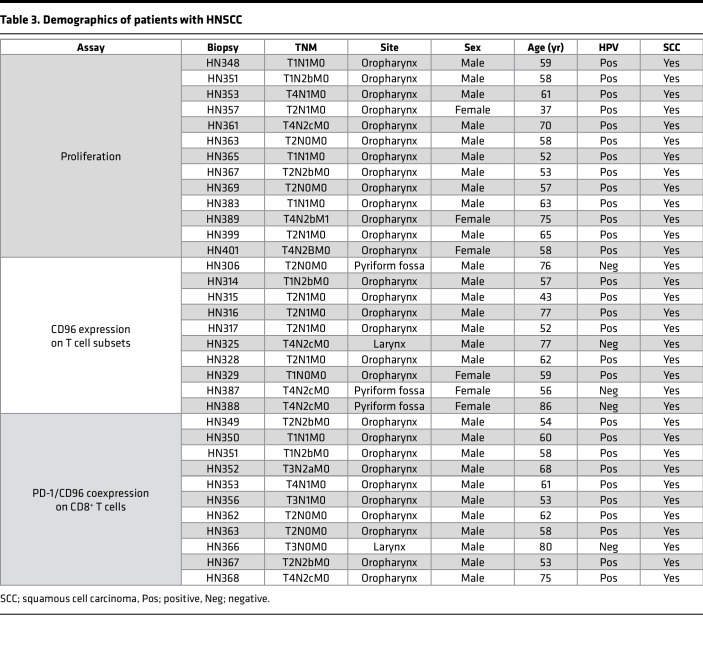
Demographics of patients with HNSCC

## Version 1 08/23/2022

In-Press Preview

## Version 2 10/10/2022

Electronic publication
