# Supplementary material for: Fcγ receptor–mediated cross-linking codefines the immunostimulatory activity of anti-human CD96 antibodies
Source: JCI Insight. 2022 Oct 10;7(19):e158444. doi: 10.1172/jci.insight.158444 (PMC9675451; doi:10.1172/jci.insight.158444)
Supplement: Supplemental data [file jciinsight-7-158444-s220.pdf]

Supplemental Figures

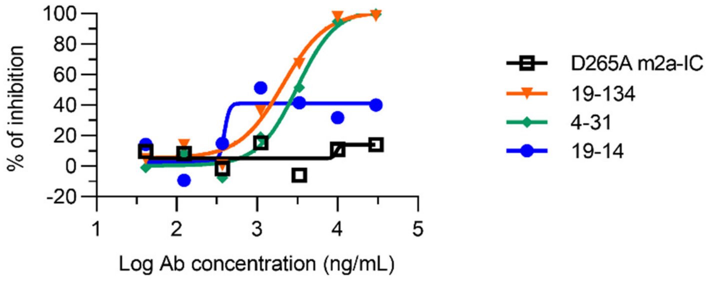

Supplemental Figure 1. CHO cell transfectants expressing human CD96 were incubated with biotinylated CD155-Fc and titrated amounts of D265A m2a anti-CD96 Ab clones or an isotype control (IC). Biotinylated CD155-Fc binding was detected using fluorescently-labelled streptavidin and flow cytometry. Data are shown as the percentage of inhibition of CD155-CD96 interaction. Dose response curves were obtained from GraphPad Prism software using a non-linear regression fit with a variable slope model.

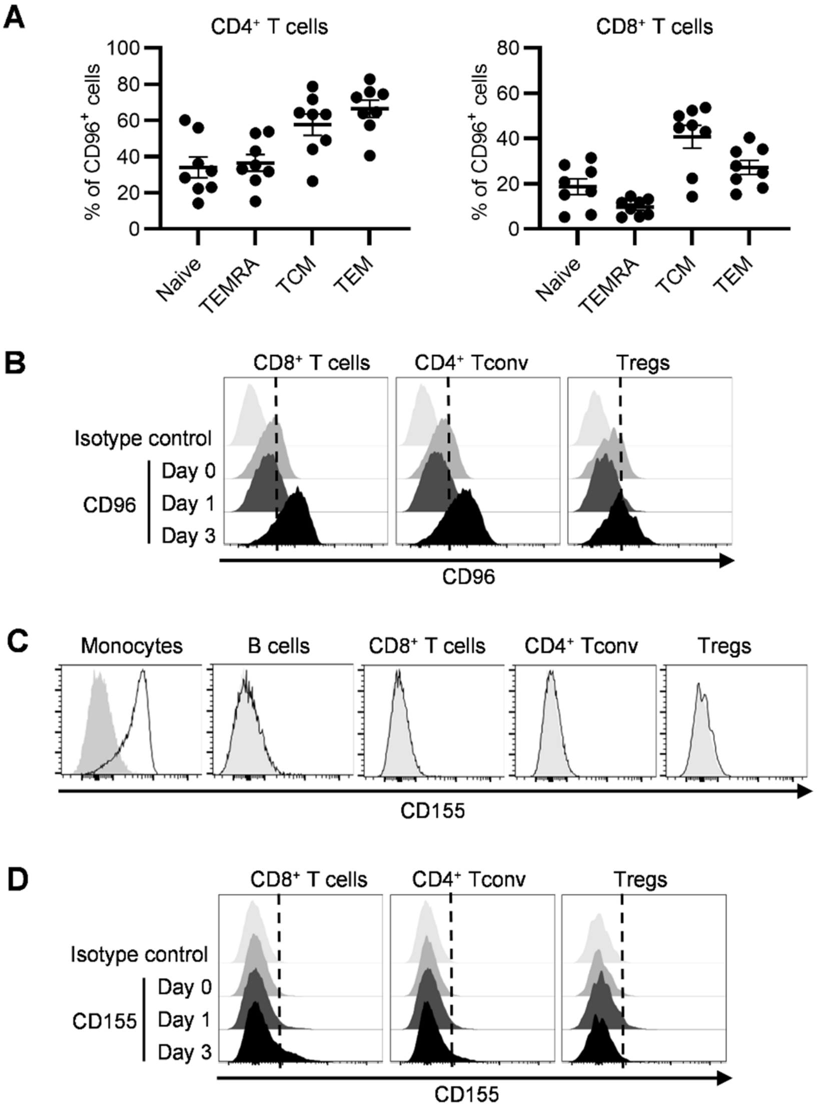

Supplemental Figure 2. The expression of CD96 and CD155 on resting and activated PBMCs from HD was analyzed by flow cytometry. **(A)** Frequency of CD96$^+$ cells among resting naive (CCR7^{hi} CD45RA^{hi}), effector memory (TEM; CCR7^{lo} CD45RA^{lo}), central memory (TCM; CCR7^{hi} CD45RA^{lo}) and terminally-differentiated memory (TEMRA; CCR7^{lo} CD45RA^{hi}) CD4$^+$ and CD8$^+$ T cells. Data show the mean ± SEM, with each symbol representing an individual HD. **(B)** Data show representative examples of CD96 expression on T-cell subsets, prior to and 1 and 3 days after stimulation with soluble OKT3. **(C** and **D)** Data show representative examples of **(C)** CD155 expression (black lines) or the corresponding isotype control (shaded histograms) on myeloid and lymphoid subsets prior to OKT3 stimulation and **(D)** of CD155 expression on T-cell subsets prior to and 1 and 3 days after soluble OKT3 stimulation. **(B-D)** Data are from one HD representative of 4, analyzed in n=2 independent experiments.

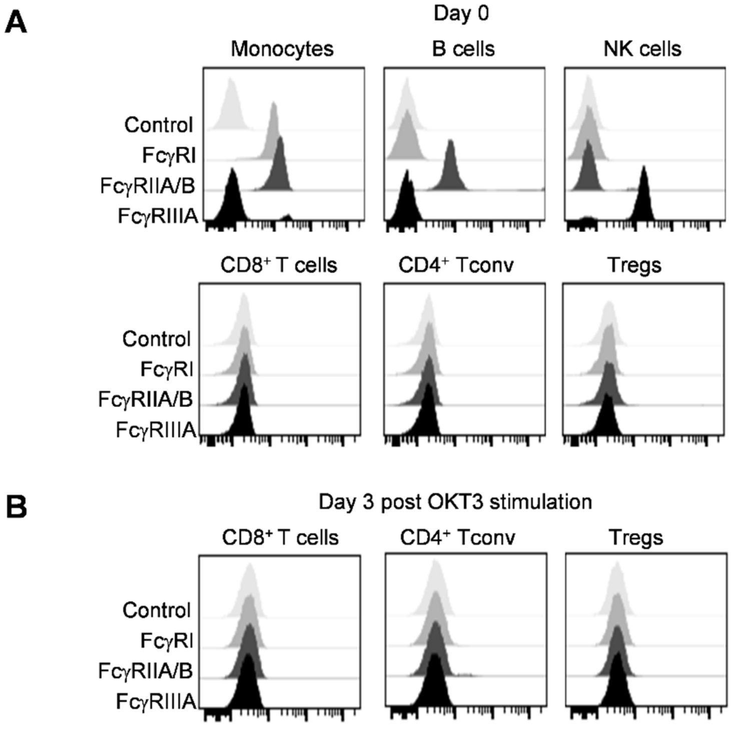

Supplemental Figure 3. The expression of FcγRI, FcγRIIA/B and FcγRIIIA on monocytes, B cells, NK cells, and T-cell subsets was analyzed by flow cytometry on PBMCs from HD, either **(A)** unstimulated or **(B)** stimulated for 3 days with soluble OKT3. Data show examples of expression for one HD representative of 4 for monocytes, B cells and NK cells, and representative of 6 for T-cell subsets, from n=3 independent experiments.

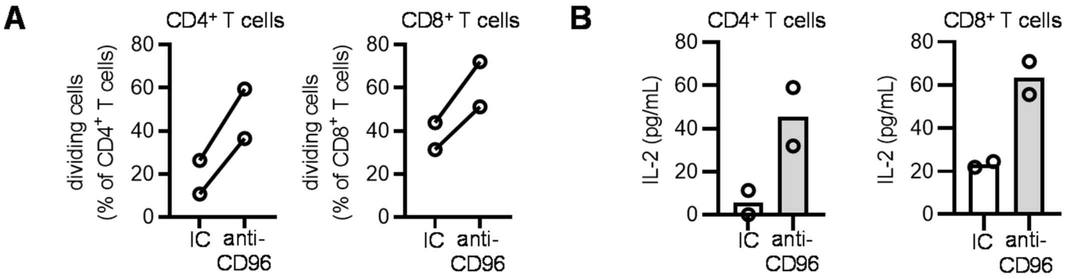

Supplemental Figure 4. T-cell subsets were purified from PBMCs from HD by immunomagnetic selection. **(A)** CFSE-labelled $CD4^+$ or $CD8^+$ T cells were stimulated with plate-bound OKT3 and plate-bound anti-CD96 huG1 mAb (19-134) or a matching isotype control (IC) for 4 days and the frequency of dividing cells was determined by flow cytometry. **(B)** $CD4^+$ and $CD8^+$ T cells were stimulated for 6 hours with plate-bound OKT3 and 19-134 huG1 mAb and IL-2 production was quantified by ELISA. Each data point represents the mean of triplicate wells for an individual donor.

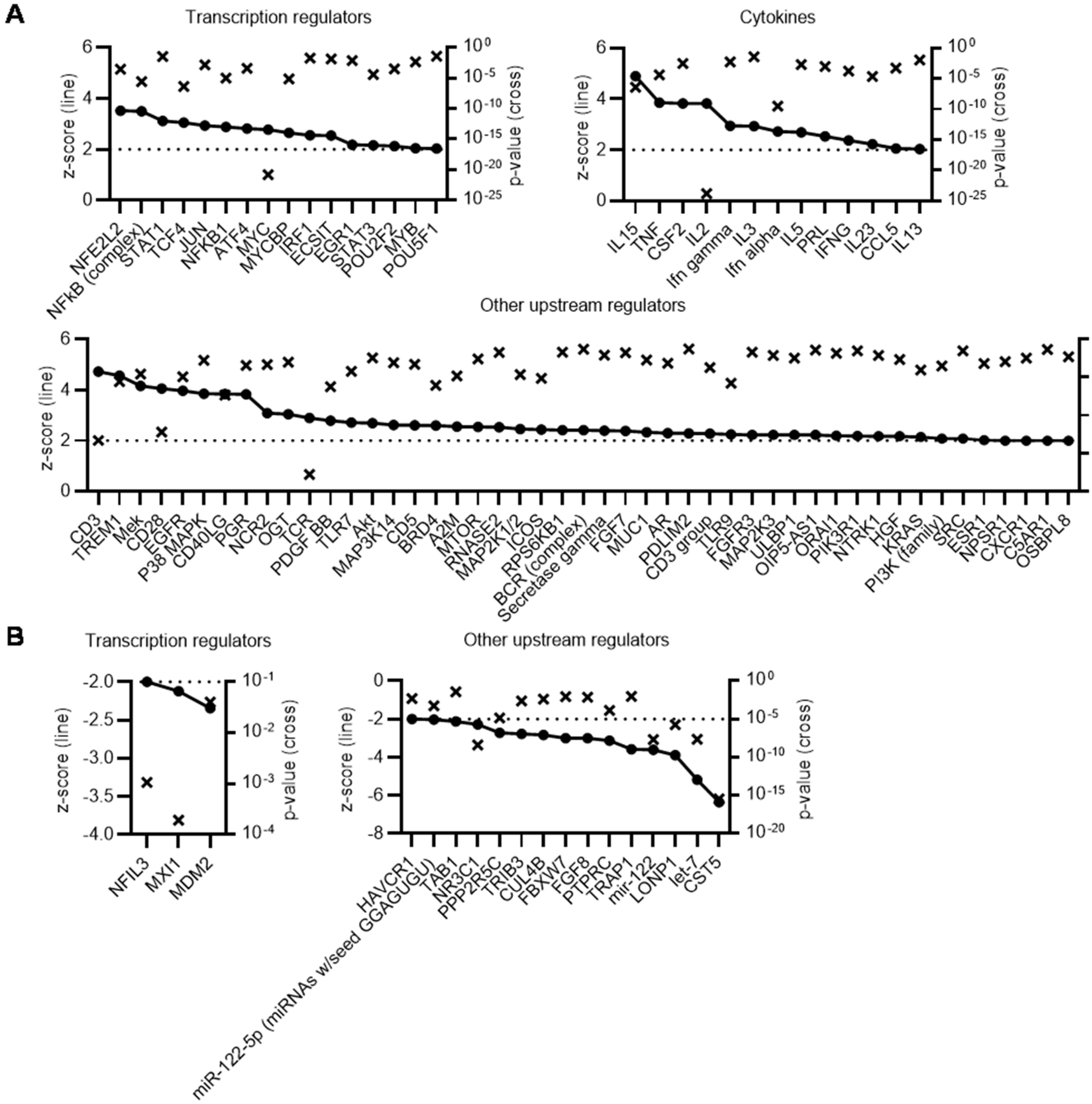

Supplemental Figure 5. Purified CD3+ T cells were stimulated for 6 hours with plate-bound OKT3 and 19-134 huG1 mAb clone or an isotype control, RNA was isolated and samples were submitted to RNA-Seq analysis. Data show upstream regulators of genes that are predicted to be **(A)** activated or **(B)** inhibited by anti-CD96 mAb, as identified by IPA; p values (crosses; right axis) and z-scores (line; left axis). Regulators with z-scores ≥ 2 are predicted to be activated; regulators with z-scores ≤ -2 are predicted to be inhibited.
